# Do Emotion Dysregulation, Alexithymia and Personality Dimensions Explain the Association Between Attention-Deficit/Hyperactivity Disorder and Binge Eating Among Bariatric Surgery Candidates?

**DOI:** 10.3389/fpsyg.2021.745857

**Published:** 2021-11-19

**Authors:** Sarah El Archi, Paul Brunault, Arnaud De Luca, Samuele Cortese, Régis Hankard, Céline Bourbao-Tournois, Nicolas Ballon, Christian Réveillère, Servane Barrault

**Affiliations:** ^1^Qualipsy, EE 1901, Université de Tours, Tours, France; ^2^CHRU de Tours, Service d’Addictologie Universitaire, Équipe de Liaison et de Soins en Addictologie, Tours, France; ^3^INSERM U1253 Imagerie et Cerveau (iBrain), Tours, France; ^4^CHRU de Tours, Centre Spécialisé de l’Obésité, Tours, France; ^5^Inserm U1069 Université de Tours, Tours, France; ^6^Academic Unit of Psychology, Center for Innovation in Mental Health, University of Southampton, Southampton, United Kingdom; ^7^Clinical and Experimental Sciences (CNS and Psychiatry), Faculty of Medicine, University of Southampton, Southampton, United Kingdom; ^8^Solent NHS Trust, Southampton, United Kingdom; ^9^Hassenfeld Children’s Hospital at NYU Langone, New York University Child Study Center, New York, NY, United States; ^10^Division of Psychiatry and Applied Psychology, School of Medicine, University of Nottingham, Nottingham, United Kingdom; ^11^CHRU de Tours, Service de Chirurgie Digestive et Endocrinienne, Chambray-lès-Tours, France; ^12^CHRU de Tours, Service d’Addictologie Universitaire, Centre de Soins d’Accompagnement et de Prévention en Addictologie d’Indre-et-Loire (CSAPA-37), Tours, France; ^13^Laboratoire de Psychopathologie et Processus de Santé, Université Paris Descartes, Paris, France

**Keywords:** addictive-like eating, binge eating disorder, ADHD, impulsivity, personality dimensions, emotion dysregulation, bariatric surgery, addictive disorders

## Abstract

**Background:** Addictive-like eating and attention-deficit/hyperactivity disorder (ADHD) are both common among persons seeking treatment for severe obesity. Given that ADHD and addictive-like eating, especially binge eating (BE) and food addiction (FA), are both strongly associated with personality dimensions and emotion dysregulation, it is possible emotional and personality characteristics contribute to the link between addictive-like eating behaviors and ADHD in people with severe obesity. This study aimed to investigate the psychological factors associated with BE and FA in bariatric surgery candidates, and to explore the mediational role of emotional factors (emotion dysregulation and alexithymia) and personality dimensions in the association between ADHD and BE.

**Method:** Two hundred and eighty-two (*n* = 282) bariatric surgery candidates were recruited during the systematic preoperative psychiatric assessment (University Hospital of Tours, France). We assessed significant BE (Binge Eating Scale), probable adult ADHD (Wender Utah Render Scale and Adult ADHD Self-Report Scale), FA (Yale Food Addiction Scale 2.0, YFAS 2.0), emotion dysregulation (Difficulties in Emotion Regulation Scale-16), alexithymia (Toronto Alexithymia Scale-20) and personality dimensions (Big Five Inventory). Mediation analyses were performed using the PROCESS macro for IBM SPSS Statistics 22.

**Results:** Prevalence of probable adult ADHD, significant BE and FA were 8.2, 19.1, and 26.6%, respectively. Participants who screened positive for addictive-like eating showed higher prevalence of probable adult ADHD, as well as higher scores on adult and childhood ADHD symptoms. They also reported lower conscientiousness, but higher emotion dysregulation, higher alexithymia, and higher neuroticism. Only BE (as opposed to FA) was also associated with lower scores on agreeableness and openness. Analysis of the association between adult ADHD and BE suggests that emotion dysregulation, conscientiousness, agreeableness, and neuroticism are total mediators and alexithymia a partial mediator.

**Conclusion:** Our findings suggest a significant association between ADHD and addictive-like eating among bariatric surgery candidates, and also suggest a significant role of emotion dysregulation and personality dimensions in this association. For individuals with ADHD and obesity, eating may be a way to cope with negative emotions, potentially increasing the risk for addictive-like eating behavior.

## Introduction

As reported in several reviews (Cortese et al., 2016; Nigg et al., 2016; Cortese and Tessari, 2017; Cortese, 2019; Ravi and Khan, 2020), Attention-Deficit/Hyperactivity Disorder (ADHD) is strongly associated with obesity, but the underlying mechanisms are still unclear (Hanć and Cortese, 2018). ADHD is a neurodevelopmental disorder defined as a persistent pattern of inattention and/or hyperactivity-impulsivity that interferes with functioning or development (American Psychiatric Association [APA], 2013). It affects 5–7% of children (Polanczyk et al., 2007; Thomas et al., 2015) and 2.5% of adults (Song et al., 2021). Impairing symptoms persist in adulthood in approximately 65% of cases (Faraone et al., 2006). According to Cortese and Tessari (2017), there is an association between obesity and ADHD in both children and adults; individuals with ADHD show a higher prevalence of obesity, and individuals with obesity show a higher prevalence of ADHD, indicating that the relationship is bidirectional. Several hypotheses can be put forward to explain this association. Obesity and associated factors lead to ADHD symptoms, ADHD and obesity share common neurobiological dysfunction, and ADHD symptoms such as impulsivity and inattention contribute to obesity, and are associated with a disruption of the circadian rhythm (Cortese and Tessari, 2017; Ravi and Khan, 2020). Ravi and Khan (2020) hypothesize the association between ADHD and obesity may be caused by disordered eating, especially binge eating (BE). The link between obesity and ADHD is relevant as the management of ADHD, both pharmacological (Cortese, 2020) and non-pharmacological (Nimmo-Smith et al., 2020) may improve the outcome of obesity (Cortese and Castellanos, 2014).

According to the 5th version of the Diagnostic and Statistical Manual of Mental Disorders (DSM-5; American Psychiatric Association [APA], 2013), binge eating disorder (BED) is characterized by eating, in a discrete period of time (e.g., within any 2-h period), an amount of food that is definitely larger than what most people would eat in a similar period of time under similar circumstances. This episode is associated with a lack of control over eating, significant distress, eating much more rapidly than normal, and eating until feeling uncomfortably full. According to Vainik et al. (2019), uncontrolled eating is associated with three psychological traits recruiting three distinct neural circuits. The first component is “high reward sensitivity,” linked to the dopamine circuit from the ventral tegmental area to the nucleus accumbens. This leads to repeated food intake to experience pleasant affect, and is especially correlated with the extraversion personality trait (Blain et al., 2020). The second component is “low cognitive control,” linked to the anterior insula and prefrontal areas. It is defined as an inability to moderate the behavioral response to heightened reward sensitivity (Vainik et al., 2019), which is particularly impaired in individuals who overeat, increasing the risk of obesity. According to Davidson et al. (2019), “weaknesses in the ability to suppress the retrieval of memories or thoughts of food, or to shift attention away from those memories, thoughts or food-related environmental cues should be associated with a higher incidence of impulsive eating behavior and excess body weight.” Moreover, Kollei et al. (2018) suggested that the effect of impaired impulse control on decision-making is associated with obesity and risk-taking, making it possible to distinguish between individuals with obesity who do and do not binge eat. For this reason, uncontrolled eating correlates positively with impulsiveness and negatively with conscientiousness, a personality trait associated with self-management and impulse control (Vainik et al., 2019). The third component is high negative emotionality, linked to the amygdala, hippocampus and the hypothalamic-pituitary-adrenal axis. As with substance use disorder (Lyvers et al., 2019), several publications have highlighted the emotional factor of food intake and overeating as a maladaptive strategy to cope with negative effects. A review conducted by Dingemans et al. (2017) indicated associations between BE, low level of mood and depressive symptoms. They suggested that individuals with BE are particularly sensitive to negative stressors, are less able to tolerate negative mood, show emotional difficulties (e.g., alexithymia), and interpersonal problems, leading to anger and frustration. These observations highlight difficulties with emotion regulation. Indeed, individuals with BED seem to have difficulty coping with negative affectivity and tend to use maladaptive strategies such as emotion suppression rather than more efficient strategies such as reappraisal.

Around 60% of individuals with BED also meet criteria for food addiction (FA) (Gearhardt et al., 2012; Ivezaj et al., 2016). Some authors argue that assessment of BED and FA provide complementary information about eating behavior (Gearhardt et al., 2012). The concept of FA is in fact inspired by DSM-5 criteria for substance-related disorders. It involves not only a lack of control over eating but also preoccupation with food, food craving, tolerance, withdrawal, and persistence despite significant negative consequences.

Interestingly, ADHD is linked to addictive-like eating behavior, such as high reward sensitivity, low cognitive control and high negative emotionality. Individuals with ADHD symptomatology are particularly at risk of addictive-like eating (Nazar et al., 2016; Capusan et al., 2017; Romo et al., 2018; Brunault et al., 2019), but the exact nature of the relationship remains unclear. Ziobrowski et al. (2018) suggested that the association between ADHD and eating disorder may in part be due to psychiatric comorbidities. Given that ADHD is strongly associated with personality dimensions such as high neuroticism (Nigg et al., 2002) and emotion dysregulation (Corbisiero et al., 2013), and that BE and FA are also associated with emotion dysregulation (Brunault et al., 2016a; Dawes et al., 2016; Benzerouk et al., 2018; Kukk and Akkermann, 2020), emotional eating (Brunault et al., 2016a; Benzerouk et al., 2018) and depression (Gearhardt et al., 2012), we hypothesized that individuals with ADHD may develop more addictive-like eating behavior due to greater emotion dysregulation and negative affectivity (El Archi et al., 2020). Hence, addictive-like eating behavior may be a way for people with ADHD to cope with emotion dysregulation, potentially increasing the risk of BE, FA and severe obesity. Initial support to this hypothesis has been provided in a study reporting that emotion dysregulation may mediate the effects of negative affectivity on BE (Kukk and Akkermann, 2020). Further support comes from a previous review that suggested a mediational role of emotion dysregulation and negative affectivity in the ADHD-addictive-like eating relationship (El Archi et al., 2020), and from a previous study highlighting the mediational role of mood and feelings in the association between inattention symptoms of ADHD and risk of disordered eating (Martin et al., 2020). Overall, investigating the association between ADHD, emotional factors, personality dimensions, and disordered eating may lead to important clinical implications for the management of people at risk of addictive-like eating. One particular population of individuals with addictive-like eating is represented by patients who are candidate to bariatric surgery.

Bariatric surgery began in the middle of the twentieth century and involves the surgical treatment of severe obesity coupled with multidisciplinary care to help patients lose weight, leading to a reduction in overall mortality (Sjöström et al., 2007) and improving quality of life and body image (De Zwaan et al., 2014). While this intervention is well known and fully documented, weight loss failure at 10 years is estimated between 20 and 35% (Christou et al., 2006). Further knowledge about the factors associated with surgery outcomes is thus needed. BED and addictive-like eating are prevalent among patients undergoing surgical treatment for severe obesity, ranging, respectively, from 13 to 21% (Dawes et al., 2016) and from 16.5 to 41.7% (Meule et al., 2012; Brunault et al., 2016a; Benzerouk et al., 2018; Müller et al., 2018). According to a review conducted by Sarwer and Heinberg (2020), up to 50% of these patients reported preoperative disordered eating, particularly loss of control over eating. Findings about the impact of comorbidities on weight loss differ, with some studies reporting that preoperative mental illness and BE were not associated with lower weight loss (Friedman et al., 2019; Saiki et al., 2020), and others showing that these comorbidities affected postoperative outcomes (Marek et al., 2017; Müller et al., 2019; Sarwer and Heinberg, 2020). In a study with a sample of bariatric surgery patients, Williamson et al. (2018) found a significant ADHD by emotion self-regulation interaction on weight loss. Consequently, comorbid ADHD and low emotion self-regulation skills was associated with poor weight loss after bariatric surgery. These results confirm the need to investigate ADHD and associated factors in patients seeking bariatric surgery in order to ensure optimal weight loss by improving pre- and post-operative care. However, to the best of our knowledge, no studies have investigated the mediational role of psychological factors in the association between ADHD and addictive-like eating in a clinical population of bariatric surgery candidates.

The aim of this study was thus to investigate for the first time the association between adult ADHD and BE in the specific population of bariatric surgery candidates. More specifically, we aimed to explore the mediational role of emotional factors (emotion dysregulation and alexithymia) and personality dimensions in this association. Secondly, we investigated the sociodemographic, weight-related and psychological factors associated with adult ADHD and addictive-like eating (i.e., BE or FA) in this population.

We expected that BE and FA would be positively and significantly associated with emotion dysregulation and alexithymia, as well as with some specific personality dimensions (i.e., high neuroticism, high conscientiousness, and low extraversion). We also expected that these variables would have a mediation effect in the association between adult ADHD and BE. Secondly, we hypothesized that ADHD would be significantly associated with a higher prevalence of addictive-like eating. We also expected that both ADHD and addictive-like-eating behavior would be associated with high levels of emotion dysregulation, alexithymia, and neuroticism and with low levels of extraversion and conscientiousness.

## Materials and Methods

### Participants and Procedure

We included all consecutive bariatric surgery candidates seen in the Nutrition Department of the University Hospital of Tours, France between July 2016 and December 2020. All data were collected during the systematic preoperative psychiatric assessment. Participants’ height and weight were directly measured to assess body mass index (BMI). They completed several self-administered questionnaires providing information about age, gender, marital and professional status, and assessing addictive-like eating behavior (BE and FA), childhood and adulthood ADHD symptoms, emotional factors (emotion dysregulation and alexithymia) and personality dimensions. Inclusion criteria were: age > 18 years, referral for a psychiatric assessment prior to bariatric surgery, and sufficient French reading proficiency. At the end of the self-administered battery, we asked participants to specify their level of comprehension of the questionnaires. The question was the following: “*How difficult was it for you to understand the questions*?” Not at all (0), A little (1), Sometimes (2), Often (3), Very often (4).” We excluded all participants who answered “often” or “very often.” Thus, we excluded patients who had difficulty understanding the questionnaire (*n* = 23) or did not complete the questionnaire in its entirety (*n* = 38). Out of 343 eligible patients, we finally recruited 282 participants, of whom 76.6% (*n* = 216) were women, with a mean age of 43.1 ± 11.2 years and mean BMI of 45.4 ± 7.7 kg/m^2^ (Table 1).

**TABLE 1:** Descriptive statistics of the whole sample and logistic regression depending on FA and significant BE status.

	Mean or %	SD or (n)	Significant binge eating			Food addiction		
Variables			OR	95% CI for OR		*OR*	95% CI for OR	
				Lower	Upper		Lower	Upper
Age (years)	43.11	11.22	1.002	0.976	1.029	1.003	0.979	1.027
Gender (% female)	76.6	(116)	0.844	0.426	1.671	1.308	0.684	2.500
Marital status (% couple)	27.7	(78)	1.839	0.874	3.871	1.279	0.695	2.354
Professional status (% professional activity)	61.3	(173)	1.186	0.645	2.180	1.436	0.837	2.463
BMI (kg/m^2^)	45.42	7.74	1.007	0.970	1.045	1.034	1.001	1.068
Significant BE*^a^*	11.23	7.87	−	−	−	1.212	1.154	1.272
Food addiction diagnosis*^b^*	2.68	2.91	1.654	1.454	1.880	−	−	−
Childhood ADHD symptoms*^c^*	21.69	17.41	1.044	1.026	1.062	1.039	1.022	1.055
Adult ADHD symptoms*^d^*	1.28	1.35	1.708	1.373	2.125	1.605	1.314	1.959
Probable adult ADHD	8.2	(23)	3.759	1.550	9.116	4.131	1.726	9.883
Emotion dysregulation*^e^*	12.49	12.54	1.072	1.047	1.098	1.055	1.032	1.078
Alexithymia*^f^*	51.31	12.34	1.052	1.024	1.080	1.029	1.006	1.052
**Personality dimensions*^g^***								
Extraversion	26.13	6.75	0.956	0.914	1.001	0.966	0.928	1.005
Agreeableness	41.45	6.12	0.939	0.888	0.994	0.954	0.907	1.004
Conscientiousness	34.86	5.92	0.861	0.814	0.911	0.907	0.866	0.951
Neuroticism	23.08	6.51	1.115	1.059	1.175	1.103	1.042	1.167
Openness	34.54	5.83	0.922	0.875	0.971	0.993	0.957	1.031

*^a^Assessed by the BES (Binge Eating Scale).*

*^b^Assessed by the YFAS 2.0 (Yale Food Addiction Scale 2.0).*

*^c^Assessed by the WURS-25 (Wender Utah Render Scale-25).*

*^d^Assessed by the ASRS (Adult Self Report rating Scale).*

*^e^Assessed by the DERS-16 (Difficulties in Emotion Regulation Scale-16).*

*^f^Assessed by the TAS-20 (Toronto Alexithymia Scale-20).*

*^g^Assessed by the BFI: Big Five Inventory; SD, standard deviation; OR, odd ratio; CI, confidence interval; BMI, body mass index; ADHD, attention-deficit/hyperactivity disorder.*

### Measures

#### Binge Eating

The 16 items of the French version of the Binge Eating Scale (BES) assess behavior, thoughts emotional states and cognitive symptoms associated with significant BE in patients with obesity (Gormally et al., 1982; Brunault et al., 2016b). Each item is a group of 4 sentences describing increasing severity of behavioral manifestations in a specific eating situation; participants choose the statement that best matches their current situation. The total score is the sum of the scores for each item and ranges from 0 to 46. A threshold of 18 was applied to indicate significant BE behavior. In the current study, the internal consistency coefficient (Cronbach’s alpha) was 0.87.

#### Food Addiction

The Yale Food Addiction Scale 2.0 (YFAS 2.0) was created to assess and diagnose FA, by extrapolating the DSM-5 substance-related and addictive disorder criteria to food (Gearhardt et al., 2016). For the current study, we used the validated French version of the YFAS 2.0 (Brunault et al., 2017). This 35-item scale concerns food behavior over the previous 12 months and assesses the clinically significant impairment or distress associated with consumption of high fat/high sugar foods. Participants respond on a Likert scale ranging from “Never” (0) to “Every day” (7). Each item refers to one of the 11 criteria of addiction. The YFAS 2.0 total score is the number of positive FA criteria for a given individual (ranging from 0 to 11) (in the current study, Cronbach’s alpha was 0.94). It is used as a dimensional and categorical variable. FA diagnosis is based on the presence of at least 2 positive criteria and clinically significant impairment or distress associated with food behavior. The severity of FA is measured by the number of positive criteria: 2 or 3 indicate mild FA, 4 or 5 indicate moderate FA, and 6 or more indicate severe FA. The French version of the YFAS 2.0 has good internal consistency: in the current study, the internal consistency coefficient for binary variables (Kuder-Richardson-20 coefficient) was 0.86.

#### Attention-Deficit/Hyperactivity Disorder

##### Childhood Attention-Deficit/Hyperactivity Disorder *Symptoms*

The Wender Utah Render Scale-25 (WURS-25) retrospectively assesses childhood symptoms of ADHD and related problems (Ward et al., 1993). The French version of the WURS-25 (Caci et al., 2010) used in the current study has 25 items scored on a 5-point Likert scale from “not at all/very slightly” (0) to “very much” (4). The total score is the sum of the scores for the 25 items, giving a possible range of 0–100. This score increases with severity of childhood ADHD symptoms. A threshold of 46 was applied to identify participants with significant childhood ADHD symptoms. In the current study, Cronbach’s alpha was 0.92.

##### Adult Attention-Deficit/Hyperactivity Disorder Symptoms

The Adult ADHD Self-Report Scale V1.1 (ASRS) is a self-report screening test of ADHD symptoms in adults, developed by the World Health Organization (Kessler et al., 2005). This widely used 6-item scale (available in several languages) assesses inattention (4 items) and hyperactivity/impulsivity (2 items). Each item is rated from “never” (0) to “very often” (4). The total score ranges from 0 to 24 and increases with severity of adult ADHD symptoms. High scores on 4 out of 6 items indicate significant adult ADHD symptoms. In the current study, we used the French version of the ASRS (Caci et al., 2008), with a Cronbach’s alpha of 0.70.

Adult ADHD criteria state symptoms include disturbance in attention and or hyperactivity/impulsivity in both childhood (before 12 years old) and adulthood. Thus, in this study, we identified “probable adult ADHD” based on screening positive both for childhood ADHD symptoms (WURS-25 ≥ 46) and for adult ADHD symptoms (number of items with significant severity ≥ 4).

#### Emotional Factors and Personality Dimensions

##### Difficulties in Emotion Regulation

The Difficulties in Emotion Regulation Scale (DERS) is a widely used questionnaire assessing emotion regulation difficulties. The previous version of the DERS had 36 items (Gratz and Roemer, 2004), measuring emotion regulation difficulties through 5 dimensions: non-acceptance of negative emotions, inability to engage in goal-directed behaviors when distressed, difficulty controlling impulsive behaviors when distressed, limited access to emotion regulation strategies perceived as effective, and lack of emotional clarity. For the current study, we used the French adaptation of the DERS-16, which is the brief 1-factor version of this scale (Bjureberg et al., 2016). Participants answered items on a 5-point Likert scale ranging from “almost never” (1) to “almost always” (5). Scores range from 16 to 80, with higher scores reflecting greater emotion dysregulation. The French version of the DERS-16 used in the current study has excellent internal consistency: Cronbach’s alpha was 0.94.

##### Alexithymia

The Toronto Alexithymia Scale-20 (TAS-20) is a 20-item questionnaire assessing alexithymia (Parker et al., 1993). Participants indicate their degree of agreement with each item using a 5-point Likert scale ranging from “strongly disagree” (1) to “strongly agree” (5). Higher total scores indicate greater severity of alexithymia. The French version of the TAS-20 (Loas et al., 1995) used in the current study has good internal consistency. In the current study, Cronbach’s alpha was 0.78.

##### Personality Dimensions

Based on the Big Five model of personality, the Big Five Inventory (BFI; John et al., 1991) evaluates 5 personality dimensions: openness (Originality, Open-mindedness; 10 items), conscientiousness (Constraint, Control of impulse; 9 items), extraversion (Energy, Enthusiasm; 8 items), agreeableness (Altruism, Affection; 10 items) and neuroticism (Negative affectivity, Nervousness; 8 items). Each of the 45 items is rated on a 5-point Likert scale ranging from “disagree Strongly” (1) to “agree Strongly” (5). In the current study, we used the French version of the BFI (Plaisant et al., 2010), with the following consistency coefficients: openness, Cronbach’s alpha = 0.77; conscientiousness, Cronbach’s alpha = 0.77; extraversion, Cronbach’s alpha = 0.80; agreeableness, Cronbach’s alpha = 0.65; neuroticism, Cronbach’s alpha = 0.78.

### Data Analysis

Analyses were conducted using SPSS^®^ version 22 (IBM Corp. Released 2013. IBM SPSS Statistics for Windows, Version 22.0., IBM Corporation, Armonk, NY, United States). All analyses were two-tailed; *p*-values ≤ 0.05 were considered statistically significant. Descriptive statistics included percentages for ordinal variables and means and standard deviations for continuous variables. Spearman correlation analyses were conducted between childhood and adult ADHD and BE, FA, emotion dysregulation, alexithymia, and the 5 dimensions of the BFI. As recommended in case of multi-comparisons (Curtin and Schulz, 1998), we adapted the threshold of significance (α’ = α/2 = 0.0025).

Our main objective was to identify the psychological factors that mediate the association between probable adult ADHD and significant BE. With this aim, we conducted logistic regression analysis to determine the variables associated with significant BE. Due to non-normal distribution of the data, simple. Mediation analyses (regression-based approach) were performed using the PROCESS macro (version 3.5.3) for IBM SPSS Statistics 22 (Hayes, 2012). Regression assumptions were confirmed: absence of outliers was verified, homoscedasticity was guaranteed through transformation of the dependent variables (square root) and verified by Levene’s test of equality of errors variances, because of the non-normal distribution, we used bootstrapping (5,000 resamples), and we assessed collinearity between variables by making sure that variance inflation factor (VIF) was under 5 as recommended (James et al., 2013).

In the mediation model of the effect of X on Y through M, X was “probable adult ADHD” (dichotomous variable), Y was “significant BE” (BES score) and M was the mediator variable. We conducted 2 simple mediations with emotion dysregulation (DERS-16 scores) and alexithymia (TAS-20 scores) as M variables, and a multiple mediation with 5 M variables, namely the 5 BFI dimensions. Unstandardized regression coefficients were identified: path *a* was the effect of “probable adult ADHD” on M, path *b* was the effect of M on “significant BE,” path *c* was the total effect of “probable adult ADHD” on “significant BE,” and path *c’* was the direct effect of “probable adult ADHD” on “significant BE.” The indirect effect of “probable adult ADHD” on “significant BE” was the product of *a* and *b*.

Secondly, we investigated the psychological factors associated with FA in our sample. The prevalence of FA within the whole sample was determined, as well as the prevalence of probable adult ADHD in the subgroup of participants with FA. We used logistic regressions to identify the sociodemographic, weight and psychological factors associated with FA and significant BE: age, gender, marital, and professional status, BMI, significant BE, ADHD symptomatology, emotion dysregulation, alexithymia, and personality dimensions.

## Results

### Descriptive Statistics

Table 1 presents descriptive data of the whole sample. Mean scores on the childhood and adult ADHD scales were 21.7 ± 17.4 and 1.3 ± 1.4, respectively, 9.9 and 45.7% of the participants showed significant childhood and adult ADHD symptoms, respectively, and 23 participants screened positive on both the childhood and the adult ADHD scales, with 8.2% of the participants showing probable adult ADHD. The mean BE score was 11.23 ± 7.87. The prevalence of significant BE was 19.1% (*n* = 54). Mean FA score was 2.68 ± 2.91, indicating mild severity. Prevalence of FA was 26.6% (*n* = 75). Forty-one participants (14.5%) showed both significant BE and FA.

### Addictive Like Eating (Binge Eating and Food Addiction) Associated With Attention-Deficit/Hyperactivity Disorder Symptoms in Bariatric Surgery Candidates

Both childhood and adult ADHD symptoms were positively correlated with BE (*r* = 0.32, *p* < 0.001 and *r* = 0.38, *p* < 0.001, respectively) and FA (*r* = 0.33, *p* < 0.001 and *r* = 0.35, *p* < 0.001, respectively).

The prevalence of probable adult ADHD was significantly higher for individuals with significant BE or FA than individuals without these addictive like eating behaviors (BE: 18.5 vs. 5.7%. χ^2^ = 9.57, *p* = 0.002; FA: 17.3 vs. 4.8%. χ^2^ = 11.49, *p* = 0.001). Bariatric surgery candidates with significant BE or FA had a higher risk of probable adult ADHD (BE: *odd ratio* (*OR)* = 3.759, *95% confidence interval (CI)*: 1.550–9.116; FA: OR = 4.131, 95% CI: 1726–9.883). The presence of probable adult ADHD was 24.4% (*n* = 10) among patients who showed both significant BE and FA.

### Associated Psychopathological Factors

According to the logistic regressions, significant BE was associated with higher scores on the scales assessing FA, childhood ADHD symptoms, adult ADHD symptoms, emotion dysregulation, alexithymia and neuroticism, and with lower scores on the agreeableness, conscientiousness and openness dimensions of the BFI. Odds ratios are presented in Table 1.

According to logistic regression, FA in bariatric surgery candidates was associated with higher BMI, and higher scores on childhood and adult ADHD scales, emotion dysregulation, alexithymia and the neuroticism dimension of the BFI. FA was also associated with lower scores on the conscientiousness dimension of the BFI. Odds ratios are presented in Table 1.

Bariatric surgery candidates with probable adult ADHD showed higher BE (*p* = 0.007), FA (*p* = 0.001), emotion dysregulation (*p* < 0.001), alexithymia (*p* = 0.012) and neuroticism (*p* < 0.001). They had lower scores for agreeableness (*p* = 0.002) and conscientiousness (*p* < 0.001). Details are presented in Table 2. Childhood and adult ADHD symptoms were also positively correlated with emotion dysregulation, alexithymia, and neuroticism, and negatively correlated with the conscientiousness, and agreeableness dimensions of the BFI. Extraversion was correlated only with adult ADHD. Details are presented in Table 3.

**TABLE 2:** Comparison between persons with vs. without probable adult ADHD.

	Probable adult ADHD					

	With *n* = 23		Without *n* = 259		Statistics	
Variables	Mean	SD	Mean	SD	*U*	*p*
Age (years)	40.7	12.3	43.4	11.1	2541.0	0.243
BMI (kg/m^2^)	47.0	11.8	45.3	7.3	2946.5	0.932
Significant BE*^a^*	16.1	9.8	10.8	7.5	1977.0	0.007
Food addiction*^b^*	5.3	4.0	2.4	2.7	1761.5	0.001
Childhood ADHD symptoms*^c^*	59.5	11.5	18.3	13.4	21.0	< 0.001
Adult ADHD symptoms*^d^*	2.9	1.2	1.1	1.3	968.0	< 0.001
Emotion dysregulation*^e^*	30.5	14.7	10.9	11.0	755.5	< 0.001
Alexithymia*^f^*	57.4	14.5	50.8	12.0	2042.5	0.012
**Personality dimensions*^g^***						
Extraversion	23.7	7.1	26.3	6.7	2321.5	0.079
Agreeableness	37.4	6.6	41.8	6.0	1841.0	0.002
Conscientiousness	29.1	7.2	35.4	5.5	1480.5	< 0.001
Neuroticism	30.6	4.5	22.4	6.2	870.0	< 0.001
Openness	34.2	7.6	34.6	5.7	2735.0	0.515

*^a^Assessed by the BES (Binge Eating Scale).*

*^b^Assessed by the YFAS 2.0 (Yale Food Addiction Scale 2.0).*

*^c^Assessed by the WURS-25 (Wender Utah Render Scale-25).*

*^d^Assessed by the ASRS (Adult Self Report rating Scale).*

*^e^Assessed by the DERS-16 (Difficulties in Emotion Regulation Scale-16).*

*^f^Assessed by the TAS-20 (Toronto Alexithymia Scale-20).*

*^g^Assessed by the BFI, Big Five Inventory; SD, standard deviation; BMI, body mass index; ADHD, attention-deficit/hyperactivity disorder; BE, binge eating; U, statistic of Mann-Whitney test.*

**TABLE 3:** Spearman correlation matrix between ADHD variables and psychopathological variables.

	WURS	ASRS
BES	0.322***	0.381***
YFAS 2.0	0.325***	0.347***
WURS-25	−	0.476***
ASRS	0.476***	−
DERS-16	0.552***	0.546***
TAS-20	0.307***	0.277***
BFI-O	–0.084	–0.027
BFI-C	0.314***	−0.459***
BFI-E	–0.118	−0.205***
BFI-A	−0.294***	−0.225***
BFI-N	0.474***	0.387***

***p ≤ 0.001; BES, binge eating scale; YFAS 2.0, Yale Food Addiction Scale 2.0; WURS-25, Wender Utah Render Scale-25; ASRS, Adult Self Report rating Scale; DERS-16, Difficulties in Emotion Regulation Scale-16; TAS-20, Toronto Alexithymia Scale-20; BFI, big five inventory (O, openness; C, conscientiousness; E, extraversion; A, agreeableness; N, neuroticism).

### Mediation Effect of Emotion Dysregulation, Alexithymia, and Personality Dimensions Between Probable Adult Attention-Deficit/Hyperactivity Disorder and Significant Binge Eating

Total effect of probable adult ADHD on significant BE was 0.723 (0.197–1.248; *p* = 0.007).

#### Mediation Effect of Emotion Dysregulation

**FIGURE 1:**
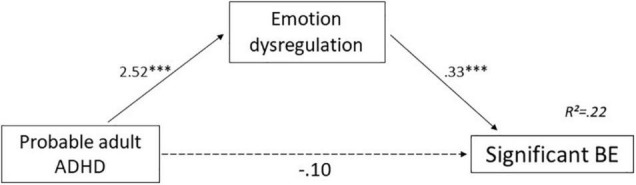
Mediation effect of emotion dysregulation in the association between probable adult ADHD and significant BE. Unstandardized regression coefficient. ^***^p< 0.001. ADHD, attention-deficit/hyperactivity disorder; BE, binge eating. Dashed arrow: Non-significant effect. Black arrow: significant effect.

Probable adult ADHD and emotion dysregulation significantly predicted BE scores [*F*(2, 279) = 38.26, *p* < 0.001; *R*^2^ = 0.22]. The direct effect of probable adult ADHD on BE (*c’*-path) was non-significant (*p* = 0.706), but effects of probable adult ADHD on emotion dysregulation (*a*-path) and of emotion dysregulation on BE (*b*-path) were significant (*p* < 0.001). Details are presented in Table 4 and Figure 1. Thus, simple mediation analysis suggests that emotion dysregulation has a total mediation effect on the association between probable adult ADHD and significant BE.

**TABLE 4:** Mediation of the effects of probable adult ADHD on significant BE.

Model	Mediators		a	b	Indirect effect a × b (95% CI)*^a^*
1	DERS-16	Emotion dysregulation	2.52***	0.33***	0.82(0.57;1.12)
2	TAS-20	Alexithymia	0.44*	0.42***	0.18(−0.00;0.40)
3	BFI-O	Openness	–0.05	–0.23	0.74(−0.06;0.09)
	BFI-C	Conscientiousness	−0.58***	−0.88***	0.51(0.23;0.88)
	BFI-E	Extraversion	–0.27	–0.04	0.01(−0.05;0.08)
	BFI-A	Agreeableness	−0.36***	0.33*	−0.12(−0.29;−0.01)
	BFI-N	Neuroticism	0.83***	0.39***	0.33(0.17;3.53)

^a^Bias corrected bootstrap results for the indirect effect, number of resamples is 5,000. *p < 0.05; ***p < 0.001.

*ADHD, attention-deficit hyperactivity disorder; BE, binge eating; DERS-16, Difficulties in Emotion Regulation Scale-16; TAS-20, Toronto Alexithymia Scale-20; BFI, big five inventory.*

#### Mediation Effects of Alexithymia

**FIGURE 2:**
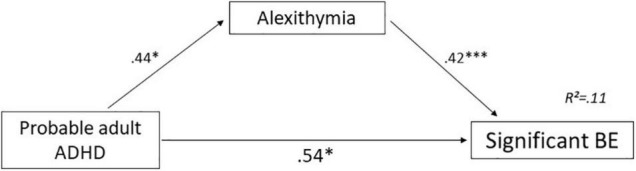
Mediation effect of alexithymia in the association between probable adult ADHD and significant BE. Unstandardized regression coefficient. *p< 0.05; ^***^p< 0.001. ADHD: attention-deficit/hyperactivity disorder; BE, binge eating. Black arrow: Significant effect.

Probable adult ADHD and alexithymia significantly predicted BE scores [*F*(2, 279) = 18.06, *p* < 0.001; *R*^2^ = 0.11]. The direct effect of probable adult ADHD on significant BE (*c’*-path) was significant (*p* = 0.037). Moreover, there were significant effects of probable adult ADHD on alexithymia (*a*-path, *p* = 0.023) and of alexithymia on BE (*b*-path, *p* < 0.001). Details are presented in Table 4 and Figure 2. These results thus suggest that alexithymia is a partial mediator in the association between probable adult ADHD and significant BE.

#### Mediation Effects of Personality Dimensions

Probable adult ADHD and the 5 dimensions of the BFI significantly predicted BE scores [*F*(6, 275) = 14.88, *p* < 0.001; *R*^2^ = 0.25]. The c’-path direct effect was not significant: –0.022 (–0.53 to 0.49), *p* = 0.933.

Openness and extraversion showed a non-significant indirect effect (*a* × *b*-path) on the association between probable adult ADHD and significant BE. The effects of openness and extraversion on BE were not significant (*b*-path; –0.23, *p* = 0.105 and –0.04, *p* = 0.710, respectively), and probable adult ADHD did not predict these dimensions (*a*-path; –0.05 *p* = 0.667 and –0.27, *p* = 0.061, respectively).

**FIGURE 3:**
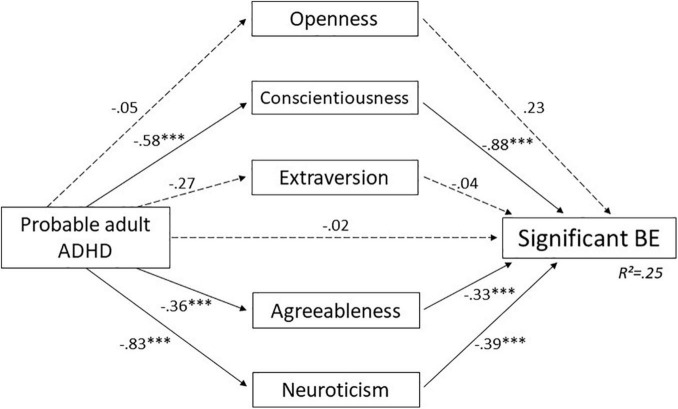
Mediation effect of personality dimensions in the association between probable adult ADHD and significant BE. Unstandardized regression coefficient. ^***^p< 0.001. ADHD, attention- deficit/hyperactivity disorder; BE, binge eating. Dashed arrow: Non-significant effect. Black arrow: Significant effect.

Conscientiousness, agreeableness, and neuroticism showed a significant indirect effect (*a* × *b*-path) of probable adult ADHD on significant BE. They were predictable by probable adult ADHD (*a*-path; –0.58 *p* < 0.001, –0.36, *p* < 0.001, and 0.83, *p* < 0.001, respectively) and had an effect on significant BE (*b*-path; –0.88, *p* < 0.001, 0.33, *p* = 0.04 and 0.39, *p* < 0.001, respectively). The results suggest a total mediational role of conscientiousness, agreeableness and neuroticism in the association between probable adult ADHD and significant BE. Details are presented in Table 4 and Figure 3.

## Discussion

This study investigated for the first time the association between ADHD and addictive-like eating in bariatric surgery candidates and investigate the possible involvement of emotion dysregulation and personality dimensions in this association. We found that BE and FA were strongly associated with ADHD as well as with specific personality dimensions (low conscientiousness and agreeableness and high neuroticism) and emotional factors (i.e., emotion dysregulation, alexithymia). We hypothesized that extraversion would have a negative effect on this association, and the results showed a non-significant effect. To the best of our knowledge, there is no published study suggesting the mediational role of emotional factors and personality dimensions in the association between ADHD and BE within a clinical population of bariatric surgery patients. The current study investigated these potential mediators, and the results suggest that emotion dysregulation, conscientiousness, agreeableness and neuroticism are total mediators in the association between ADHD and BE, and that alexithymia is a partial mediator.

The prevalence of probable adult ADHD in our sample of bariatric surgery candidates was estimated to be 8.2%. As expected, this is higher than in the general population (Song et al., 2021), estimated at around 2.55. Prevalence increased to 17.3–18.5% when the analysis included only patients with BE or FA. These results are consistent with prior studies conducted by Alfonsson et al. (2013) and Nielsen et al. (2017) with the same population, with estimated ADHD prevalence of 8.3 and 8.6% respectively. Most of the literature reports that disordered eating involving binging/purging behavior is associated with a higher prevalence of ADHD symptomatology, varying between 12 and 37.1% (El Archi et al., 2020).

Our results support the hypothesis of a positive correlation between ADHD and addictive-like eating. Studies have provided genetic, neurobiological and behavioral evidence of the association between ADHD symptoms and reward sensitivity (Luman et al., 2010; Carey et al., 2017). The combination of reward sensitivity, sensation seeking and difficulty with impulse control encourages instant gratification, despite the negative consequences. In this way, all behaviors involving positive sensations are risky in that they can lead to a desire to repeat a pleasurable experience and potentially addiction. This is heightened by high cue-reactivity shown by individuals with ADHD (Vollstädt-Klein et al., 2020). Previous publications have suggested that this pattern can also be observed in substance-use disorders such as problematic alcohol use (Vollstädt-Klein et al., 2020), and it seems to be compatible with food intake and addictive-like eating behavior.

Mediation analyses support the hypothesis that conscientiousness, agreeableness, and neuroticism are total mediators in the association between adult ADHD and BE; neuroticism had a positive effect in this association, while conscientiousness, and agreeableness had a negative effect. John and Srivastava (1999) described these three traits as follows: Conscientiousness involves socially prescribed impulse control that facilitates task- and goal-directed behavior, such as thinking before acting, delaying gratification, following norms and rules, and planning, organizing, and prioritizing tasks; agreeableness contrasts a prosocial and communal orientation toward others with antagonism, and includes traits such as altruism, tender-mindedness, trust, and modesty; and neuroticism contrasts emotional stability and even-temperedness with negative emotionality, such as feeling anxious, nervous, sad, and tense. Previous publications have shown that addictive disorders (both substance use disorders and gambling disorder) are particularly associated with high neuroticism and low conscientiousness and agreeableness (Dash et al., 2019), suggesting that poor social abilities, negative affect and impulsivity are vulnerability factors for addictive disorders. Moreover, personality traits seem to be involved in addictive disorders; Davis et al. (2015) observed that personality traits frequently found in individuals with ADHD, such as impulsivity, reward drive and neurotic traits, may underlie the positive association between ADHD symptomatology and addictive behaviors, suggesting that these personality traits drive their proneness to engage in immediately reinforcing activities. Impulsivity, defined as the tendency to act rashly when experiencing strong emotions (urgency) and high levels of delayed reward discounting, are associated with food addiction (VanderBroek-Stice et al., 2017). Moreover, food addiction seems to be a mediator between these impulsivity dimensions and obesity. According to previously cited authors (VanderBroek-Stice et al., 2017), individuals who tend to act rashly when feeling strong emotions may be more likely to engage in compulsive eating behavior. Moreover, according to Keller and Siegrist (2015), neuroticism and conscientiousness are, respectively, positively and negatively correlated with emotional eating, and to a lesser extent with food intake in response to external cues (external eating). They suggested that external eating and emotional eating are significantly and positively associated with sweet and savory food consumption. Both Neuroticism and Extraversion had a significant positive indirect effect on sweet and savory food consumption, whereas Conscientiousness had a significant negative indirect effect. Thus, personality traits seem to influence both eating style and the type of food eaten. This could explain our findings that neuroticism, agreeableness and conscientiousness mediate the association between ADHD and addictive-like eating, especially BE.

As expected, emotion dysregulation was a total mediator factor in the association between adult ADHD and BE. Indeed, in a study by Şahan et al. (2021), bariatric surgery candidates who screened positive for ADHD showed higher emotional eating and susceptibility to hunger than those who screened negative. Negative emotion is a trigger for food intake. Positive emotion seeking would increase when negative emotions occur, particularly if it is associated with emotion dysregulation. Our results suggest that emotion dysregulation of individuals with ADHD leads to difficulty coping with negative emotions and a tendency to avoid them. This may affect self-esteem, well-being, and social skills, increasing the risk of psychiatric comorbidities such as depression and anxiety disorders. On the other hand, we hypothesized negative emotions associated with distress may either lead to negative secondary emotional responses or may be avoided in favor of immediate positive emotion seeking and hence lead to addictive behavior and disordered eating.

Our results also suggest that alexithymia may be a partial mediator in the association between ADHD and BE. Alexithymia involves difficulty in identifying and describing emotions, including one’s own. Some individuals with ADHD show high alexithymia (Edel et al., 2010), with significantly less “acceptance of emotions,” less “experience of self-control,” more “experience of being flooded with emotions,” more “experience of lack of emotions,” and more “imaginative symbolization of emotions” (Edel et al., 2010). Moreover, women with morbid obesity scored higher on alexithymia (especially on difficulty identifying feelings) and suppression of emotions than the general population (Zijlstra et al., 2012). According to a meta-analysis conducted by Westwood et al. (2017), individuals with eating disorders have more difficulty identifying and describing emotions than those without eating disorders, suggesting that individuals with eating disorders have significant difficulty using adaptive and situationally appropriate emotion regulation strategies, such as impulse inhibition and behavioral control when distressed, emotional approach and tolerance, and particularly emotional awareness, clarity and acceptance. These difficulties share characteristics with alexithymia, and resorting to food intake or addictive behavior may be a way to deal with them (Marchetti et al., 2019). Our results are in the line with the literature which indicate that alexithymia should be included in the assessment of bariatric surgery candidates, particularly as it is associated with lower weight loss (Paone et al., 2019; Lai et al., 2021).

Our results propose clinical implications. Vulnerabilities shared by individuals with ADHD and people with addictive-like eating suggest that clinicians should pay close attention to ADHD and addictive-like eating comorbidity, both among individuals with ADHD in order to prevent BE and food addiction, and among those at high risk of disordered eating such as bariatric surgery candidates. Systematically screening bariatric surgery candidates for ADHD and addictive-like eating behavior will ensure provision of appropriate treatment. If future longitudinal studies will confirm the current transversal results of the role of emotion dysregulation, alexithymia, neuroticism, agreeableness, and conscientiousness in the association between adult ADHD and addictive-like eating, we could suggest specific psychological interventions. It could be possible to target emotion regulation by helping patients develop suitable coping strategies and flexibility, acceptance and recognition of their emotions in order to improve their management of negative affectivity, reduce their tendency to act rashly when experiencing a negative mood (negative urgency) and hence resort to compulsive eating. The total mediator role of low conscientiousness between adult ADHD and BE suggests the relevance of targeting impulsivity in therapy, encouraging delayed gratification and planning. This seems to be especially relevant for bariatric surgery candidates who show a high prevalence of ADHD symptomatology and addictive-like eating behaviors. Cognitive-behavioral therapy could be proposed, targeting emotion, cognitive distortions, behavioral compensatory skills and management of impulse control, as it has been shown to be the most effective long-term treatment of adult ADHD (López-Pinar et al., 2018). Mindfulness has also been shown to be effective in reducing BE and emotional eating by “cultivating awareness of internal experiences (e.g., emotions, physical sensations), facilitating self-acceptance, cognitive flexibility, compassion, and forgiveness, and generally improving one’s ability to cope adaptively with emotions” (Katterman et al., 2014). As suggested by Martinez-Motta et al. (2020), mindfulness meditation could increase the psychological flexibility of bariatric surgery candidates, leading to a decrease in perceived stress and an increase in intuitive eating. These kinds of pre- and post-operative psychological interventions could help reduce compulsive behavior and hence weight loss failure.

This study has a number of limitations. First, mediational analyses were conducted through a cross-sectional study. This kind of descriptive design does not allow to identify causal links, as only a longitudinal design would provide a reliable causal link. This would be particularly interesting in order to investigate the effect of childhood ADHD on emotional skills development, and on eating behavior and severe obesity in adulthood. Secondly, all data were collected through self-administered questionnaires, including assessment of ADHD. Future studies should include structured clinical interviews to assess childhood and adult ADHD and addictive-like eating behavior. Self-administered questionnaires are not enough to diagnose ADHD and would not enable us to question finer points of ADHD DSM-5 criteria, to make sure symptoms have a significant impact in different areas of everyday life, to prevent failure to understand items and memory bias. Moreover, the questionnaires used in the current study did not distinguish between inattention and hyperactivity/impulsivity symptoms, which may have masked individual differences. Gender characteristics of the participants are representative of bariatric surgery candidates. And so, females are predominant in the sample. Especially because ADHD is more prevalent in male, there is a possible gender bias. To increase individualized caring, future studies could investigate gender differences. It should be noted that the concept of food addiction is still under debate, as can be seen from the recent articles by Gearhardt and Hebebrand putting forward the pros and cons of the concept of “food addiction” (De Luca and Brunault, 2021; Gearhardt and Hebebrand, 2021; Hebebrand and Gearhardt, 2021). However, these authors agree that certain foods may lead to addictive-like eating, that mechanisms involved in substance-related and addictive disorders contribute to overeating and obesity, and that the YFAS 2.0 has good psychometric properties, is widely used by clinician and researchers, and is the best psychosocial predictor of weight-loss failure (Gearhardt and Hebebrand, 2021; Hebebrand and Gearhardt, 2021). Future studies should also investigate the effect of psychological interventions targeting emotion regulation on weight loss success following bariatric surgery, and the possible links between abnormal eating patterns and alterations in sleep and arousal, which have also been found in the relationship between ADHD and severe obesity (Cortese et al., 2008a,b).

## Conclusion

The current study provides insight into the relationship between ADHD and addictive-like eating among bariatric surgery candidates and tested the hypothesis of a specific psychological mechanism that may explain this association. Identifying the pre-surgical psychological factors associated with disordered eating in bariatric surgery candidates may help design more tailored-based interventions aimed at improving the outcome of bariatric surgery.

## Data Availability Statement

The raw data supporting the conclusions of this article will be made available by the authors, without undue reservation.

## Ethics Statement

The studies involving human participants were reviewed and approved by the institutional review board of the “Comité d’Éthique pour les Recherches Non-Interventionnelles” [CERNI] (Tours-Poitiers) in 2018 (no. 2018 048). All collected data were in line with the French recommendation regarding use of personal data, with the approval of the French Commission Nationale de l’Informatique et des Libertés (CNIL). The patients/participants provided their written informed consent to participate in this study.

## Author Contributions

AD, RH, and CB-T: data collection. SE: writing—original draft preparation. SE, PB, AD, SC, RH, CB-T, NB, CR, and SB: writing—review and editing. SB and PB: study design, concept, and supervision. All authors have read and agreed to the published version of the manuscript.

## Conflict of Interest

NB reports personal fees from Lundbeck, Astra-Zeneca and D&A Pharma, unrelated to the submitted work. PB reports personal fees and non-financial support from Lundbeck, personal fees from Astra-Zeneca and D&A Pharma, unrelated to the submitted work. SC declares honoraria and reimbursement for travel and accommodation expenses for lectures from the following non-profit associations: Association for Child and Adolescent Central Health (ACAMH), Canadian ADHD Alliance Resource (CADDRA), British Association of Pharmacology (BAP), and from Healthcare Convention for educational activity on ADHD. The remaining authors declare that the research was conducted in the absence of any commercial or financial relationships that could be construed as a potential conflict of interest.

## Publisher’s Note

All claims expressed in this article are solely those of the authors and do not necessarily represent those of their affiliated organizations, or those of the publisher, the editors and the reviewers. Any product that may be evaluated in this article, or claim that may be made by its manufacturer, is not guaranteed or endorsed by the publisher.
